# Structure is more robust than other clustering methods in simulated mixed-ploidy populations

**DOI:** 10.1038/s41437-019-0247-6

**Published:** 2019-07-08

**Authors:** Marc Stift, Filip Kolář, Patrick G. Meirmans

**Affiliations:** 1 Ecology, Department of Biology, University of Konstanz, Konstanz, Germany; 2 Department of Botany, Faculty of Science, Charles University in Prague, Prague, Czechia; 3 Department of Botany, University of Innsbruck, Innsbruck, Austria; 4 Institute for Biodiversity and Ecosystem Dynamics, University of Amsterdam, Amsterdam, The Netherlands

**Keywords:** Genetic variation, Evolutionary genetics

## Abstract

Analysis of population genetic structure has become a standard approach in population genetics. In polyploid complexes, clustering analyses can elucidate the origin of polyploid populations and patterns of admixture between different cytotypes. However, combining diploid and polyploid data can theoretically lead to biased inference with (artefactual) clustering by ploidy. We used simulated mixed-ploidy (diploid-autotetraploid) data to systematically compare the performance of *k*-means clustering and the model-based clustering methods implemented in Structure, Admixture, FastStructure and InStruct under different scenarios of differentiation and with different marker types. Under scenarios of strong population differentiation, the tested applications performed equally well. However, when population differentiation was weak, Structure was the only method that allowed unbiased inference with markers with limited genotypic information (co-dominant markers with unknown dosage or dominant markers). Still, since Structure was comparatively slow, the much faster but less powerful FastStructure provides a reasonable alternative for large datasets. Finally, although bias makes *k-*means clustering unsuitable for markers with incomplete genotype information, for large numbers of loci (>1000) with known dosage *k-*means clustering was superior to FastStructure in terms of power and speed. We conclude that Structure is the most robust method for the analysis of genetic structure in mixed-ploidy populations, although alternative methods should be considered under some specific conditions.

## Introduction

Studying population structure is of key importance for understanding patterns of gene flow and admixture and for inferring the demographic history of populations. Thanks to increased computational power, the recent decades have seen a rapid development and application of methods and software to infer population structure. The clustering algorithm implemented in Structure (Pritchard et al. 2000) is by far the most popular, but there are several alternatives. As a result, reporting population structure has become a standard element of population genetic studies. Although some of the available methods have in principle been developed for diploids, they can be and have been used for polyploids, including datasets encompassing individuals of different ploidy (mixed ploidy).

The occurrence of multiple ploidy levels within the same species likely represents the early phases of polyploid speciation. Population genetic structure in polyploid complexes can thus provide unique insights in the origin of polyploid populations and patterns of admixture within and between ploidy levels (Kolar et al. 2017). Studying structure of an established mixed-ploidy species or species group also aids inference on the recurrent origins of polyploids and strength of gene flow across the ploidy barrier. For example, Monnahan et al. (2019) used clustering methods to explore genome-wide single nucleotide polymorphism (SNP) data of *Arabidopsis arenosa* and to formulate a baseline hypothesis of inter-ploidy admixture, which was further tested by coalescent simulations. Zozomová-Lihová et al. (2015) used clustering approaches to demonstrate genetic separation between diploid and tetraploid cytotypes of *Cardamine amara*. However, the potential biases that could arise when combining diploid and polyploid data utilizing clustering methods developed for diploids, have not been investigated systematically.

A key source of potential bias is the fact that diploids and polyploids cannot be genotyped with the same precision, because of the difficulties associated with genotyping polyploids (Dufresne et al. 2014). This stems from the fact that in polyploids there is not simply one, but several heterozygous genotypes that differ in the dosage of the various alleles. For example, a di-allelic locus in a tetraploid has three different heterozygote genotypes (AAAB, AABB, and ABBB), and this number increases with increasing ploidy (pentaploids: 4; hexaploids: 5; octoploids: 7). Since it is often difficult to infer the dosage from marker phenotypes, these heterozygotes can often not be distinguished from each other. This in turn complicates accurate estimation of the allele frequencies, on which most clustering algorithms rely for inference. To circumvent such genotyping problems –as well as problems with the development of co-dominant markers—researchers have used dominant markers such as AFLPs for the analysis of polyploid species (e.g. Španiel et al. 2011; Kuzmanović et al. 2013). However, dominant markers hardly solve the problem as they simply present a further reduction of the available genotype information and potentially increases the inter-ploidy bias.

The problems with incomplete genotypes and dominant markers may become especially problematic in cases where the study species does not have a single ploidy level, but represents a mixture of different ploidies. This is because the degree of information loss due to unknown dosage inevitably increases with the ploidy level, since higher ploidy levels will have more unknown allelic states. For example, band presence in AFLP identifies the presence of a dominant allele (A), but does not inform on the number of copies of that allele. Band absence identifies absence of the dominant allele. This principle causes inherently different band frequencies between ploidy levels. For example, under HW equilibrium (with *q* representing the frequency of the recessive allele B), the recessive homozygote genotype corresponding to band absence would occur at a frequency *q*^*2*^ in a diploid population, but only at a frequency *q*^*4*^ in a tetraploid population (Moody et al. 1993). In other words, band absence becomes increasingly rare with higher ploidy. As a result, samples may cluster by ploidy rather than by actual genetic signal. This is especially problematic for empirical studies that seek to infer the number of origins of polyploidy through clustering analyses (e.g., Kolar et al. 2012; Mandak et al. 2016; Tomasello and Oberprieler 2017). Therefore, it is of critical importance to theoretically evaluate the conditions under which potential biases can arise and their severity.

Here, we use simulated data to test whether the presence of multiple ploidy levels biases the inference of population genetic structure using several clustering methods/software programs. More specifically, we ask whether *k*-means clustering (as implemented in Adegenet; Jombart (2008)) and the model-based approaches implemented in Structure (Pritchard et al. 2000), FastStructure (Raj et al. 2014), Admixture (Alexander et al. 2009), and InStruct (Gao et al. 2007) erroneously aggregate individuals according to ploidy, even when there is no divergence between ploidy levels. Furthermore, we ask whether such bias increases with the reduction of information due to unknown dosage and dominance. We do this for different levels of differentiation between two simulated mixed-ploidy populations, for different marker types and different numbers of loci and individuals.

## Methods

### Simulations

To test for possible ploidy-related bias in the clustering methods, we simulated mixed-ploidy data where there is no differentiation between ploidy levels, but there is differentiation among populations. When clustering of the simulated data shows grouping by ploidy level –rather than by population– this is a clear sign of bias in the used method. Though this approach makes the unrealistic assumption of a complete absence of a reproductive barrier between the ploidy levels, it is a useful null model that fits the purposes of this paper; our aim is to uncover potential bias, not to accurately model the effects of the genetic relationships between ploidy levels.

We simulated two populations, both containing *N* = 1000 individuals, equally split between 500 diploids and 500 autotetraploids. The modelled species is a hermaphroditic (monoecious) annual with completely random mating within populations, including the possibility of selfing. The two populations are connected by migration at rate *m*, which is varied over simulation scenarios in order to obtain a range of different strengths of population differentiation. A set of *l* biallelic selectively neutral SNP loci is simulated; all loci are independently segregating (unlinked) since this is an assumption made by most of the tested clustering methods and best represents current genotyping practices. Mutation, with possibility of reverse mutation, takes place at a rate *u*, which was set at *u* = 0.0001 for all simulation scenarios, unless stated otherwise. This value resulted in a suitable level of genetic variation at the modelled SNPs.

The model is not individual-based but instead tracks the allele frequencies at all loci in the two populations. Each population is therefore modelled as a vector of *l* integers, each representing the number of copies of one of the two alleles (A or B) present in the population. The value of each integer can range from 0 (fixation of the B allele) to 3000 (fixation of the A allele). The latter number derives from the fact that there are 500 diploids and 500 tetraploids, and therefore a total of 500*2 + 500*4 = 3000 copies of the haploid genome present in each population.

Every generation, the drawing of gametes is simulated by drawing random numbers from a binomial distribution, based on the allele frequencies stored in the vectors while allowing for migration and mutation. For every locus in every population first the expected allele frequency is calculated after mutation. For example, when the number of allele copies in a population is 3000 (so fixation), the expected frequency after mutation is 1.0 − 0.0001 = 0.9999. Then, in a similar way, this expected frequency is adjusted for the expected migration from the other population, taking into account the frequency of the allele in that population. Then a random number was drawn from a binomial distribution, using the expected frequency as the probability parameter and 3000 for the size parameter (the number of trials). In the integer vector, the number of allele copies for this locus and population was then replaced by the randomly drawn number. This was repeated for 100,000 generations, which was more than enough to reach mutation-migration-drift equilibrium. The model was created and run in *R*, heavily relying on the *rbinom*() function.

After the simulation was finished, genotypes were constructed for a sample of *n* diploid and *n* tetraploid individuals from each population, based on the vector of allele copy numbers at the final generation. From these genotypes, three different datasets were created: (1) Co-dominant data. This dataset simply contained the full diploid and tetraploid genotypes with known dosage. (2) Data with unknown dosage. For this dataset, the tetraploid individuals had dosage information removed for heterozygous loci, meaning that genotypes AAAB, AABB, and ABBB all had phenotype AB. The diploid genotypes remained unchanged. 3) Dominant data. The A allele was chosen to be dominant and the B allele recessive. The dataset was then coded as presence-absence based on the presence of the A allele (comparable to the coding of AFLP data). The three datasets were then (if necessary) written to external files in the appropriate formats for the different clustering software packages.

### Scenarios

In our default simulation scenario, we tested the effect of the strength of the population differentiation on the quality of the clustering results. For this, we used the two populations as described above with eight different migration rates ranging from *m* = 0.1 to *m* = 0.001, resulting in multilocus *F*_st_-values between 0 and 0.15. We performed 10 independent simulations for each of those migration rates. The simulated genomes contained 100 loci, each with a mutation rate of *u* = 0.0001. At the end of the simulation, 400 individuals were randomly sampled, 100 diploids and 100 tetraploids in each of the two populations. To test the effect of the level of genetic variation, this same simulation scenario was also run with a mutation rate of *u* = 0.00001.

In our second scenario, we explored the trade-off between sampling more individuals or sampling more loci. For this we selected two migration rates (*m* = 0.01 and *m* = 0.001) that resulted in relatively weak and strong population structure (average *F*_st_ -values across replicates of 0.016 and 0.12, respectively). We then varied the number of simulated loci (10, 30, 100, 300, and 1000), and the total number of sampled individuals (40, 120, and 400), simulating all pairwise combinations of number of loci and individuals (10 replicates per combination).

Finally, we simulated a scenario with a modern genotyping-by-sequencing (GBS) dataset where a limited number of individuals was genotyped at a large number of SNP-markers. For this, the total number of sampled individuals was set at 40 and the number of loci at 10,000. As in the first scenario above, eight migration rates were used between 0.1 and 0.001 (with 10 replicates each). For this scenario, we did not create a dataset with dominant markers, as such datasets do not occur in practice. Another problem of GBS datasets is undercalling: heterozygotes that are called as as homozygotes, because only one of the alleles is present in the data as a result of insufficient sequencing depth. This problem is expected to be more severe in polyploids than in diploids, since polyploids have partial heterozygotes in which it is more likely that the rare allele (e.g. the B-allele in an AAAB genotype) is not present among the sequences. However, we did not include any undercalling in our simulated datasets: preliminary simulations revealed that it does not lead to any differences in inferred allele frequencies between diploid and polyploid populations and is therefore not expected to lead to spurious clustering.

### Clustering analyses

The two simulated populations were analysed using the five clustering methods detailed below. Reflecting these two simulated populations, we set *k* = 2 (number of clusters to be inferred = 2) for all analyses. As such, we did not perform an estimation of the optimal number of clusters *k*, as this value is not informative about potential spurious clustering. For example, even if the optimal number of clusters would be (correctly) estimated as *k* = 2, this could both reflect (correct) clustering of individuals by population, or (incorrect) clustering of individuals by ploidy.

K-means is a general-purpose clustering method that attempts to find the best clustering of objects into *k* groups by optimising the among-groups sum of squares. In genetic analyses, *k*-means is used either as a stand-alone analysis of population structure, as one of the steps in a DAPC analysis (Jombart et al. 2010), or in an Analysis of Molecular Variance framework (Meirmans 2012). We used the *find.clusters*() function from the R-package Adegenet v. 2.1.1 (Jombart 2008; Jombart and Ahmed 2011) to perform the *k*-means analysis on all three datasets (co-dominant known and unknown dosage, and dominant). This function is usually executed as part of a DAPC analysis, which also includes selection of axes from a Principal Components Analysis and a Discriminant Analysis. However, we did not perform these additional analyses as they are more difficult to automate across a large number of datasets (see helpfile provided with adegenet). Therefore, the analysis as we performed it is equivalent to performing a *k*-means analysis on the matrix of within-individual allele frequencies (which can take values of 0, 0.5, and 1 in diploids, and values of 0, 0.25, 0.5, 0.75, and 1 in tetraploids).

Structure performs Bayesian assignment of individuals to a predefined number of clusters (Pritchard et al. 2000). It can accommodate data from any ploidy level and allows specifying whether dosage information is known. In addition, Structure can handle dominant data, coded as presence/absence data, with the possibility to specify the known ploidy of individuals. We ran Structure v 2.3.4 on all three datasets (co-dominant with known and unknown dosage, and dominant) using the admixture model with uncorrelated allele frequencies. The Monte Carlo Markov Chain was run for 100,000 steps, following a burn-in period of 10,000 steps. This was enough to reach convergence; trials with longer chains did not yield different results. Ten replicates were run for every analysis and the one with the highest overall likelihood was selected for parsing the results.

Admixture performs maximum likelihood estimation of ancestry of individuals based on multilocus SNP data (Alexander et al. 2009). Though it is similar to Structure in that it estimates the amount of admixture in individuals (hence its name), it is based on a different optimisation algorithm and therefore much faster. Unfortunately, Admixture only takes diploid data, but because its speed makes it attractive for the large datasets resulting from modern genotyping techniques we decided to include it anyway. For the co-dominant data, we subsampled the tetraploid genotypes by randomly sampling two of the four allele copies (cf. Novikova et al. 2016; Monnahan et al. 2019). Note that subsampling makes the tetraploids equivalent to the diploids, since the tetraploids were derived from the same gene pool to begin with. So this data can only be used to judge the performance of Admixture and not any ploidy-induced bias. However, for data with unknown dosage, ploidy-related bias is still possible because subsampling does not by-pass the problem that higher ploidies have missing information, and diploids do not. We created the dataset with unknown dosage by coding all heterozygous tetraploid genotypes as AB and the homozygous genotypes as AA or BB. It is not possible to run Admixture with dominant data. Admixture v. 1.3.0 was run with the default block relaxation algorithm, the default stopping criterion of *ε* = 10^−4^, and 5 times cross-validation.

FastStructure is an alternative to Structure that was developed especially for large SNP datasets (Raj et al. 2014). Like Admixture, FastStructure takes neither polyploid nor dominant data. Therefore, we used the same subsampling strategy as for Admixture to create the input files for FastStructure. FastStructure v 1.0 was run with the default convergence criterion of 10^−6^, a simple prior, and ten replicate runs per dataset.

InStruct is an extension of the Structure algorithm that allows simultaneous inference of the population structure and the inbreeding rate (Gao et al. 2007). Though InStruct can handle diploid or tetraploid data, the manual makes no reference to mixed-ploidy datasets or whether it allows unknown dosage information. For the input files we coded the diploid data as tetraploid with two instances of missing data. Similarly, for the dataset with unknown dosage we coded the heterozygous loci for tetraploid individuals as A, B, and two instances of missing data. Since InStruct does not support dominant data, we only ran InStruct for two out of the three datasets (co-dominant known *vs*. unknown dosage). InStruct v. 1.0 was used with 100,000 steps for the Monte Carlo Markov Chain, following a burnin of 10,000 steps. For the MODE parameter we used the default value of 1, to infer population structure only with admixture, so without estimating the inbreeding coefficient or selfing rate. Ten replicates were run for every analysis and the one with the highest overall likelihood was selected for parsing the results. Because of the exceedingly long time required for running each replicate (>6 h, whereas Structure took <30 min) and the overall inconsistent performance, we only ran InStruct for the default scenario of 100 loci and 100 sampled individuals.

### Parsing of results

R version 3.4.3 (R-Core-Team. 2017) was used to automate running the clustering analyses and for parsing the output produced by the different programs. For determining the fit of the clustering results to either the populations (correct) or the ploidy levels (bias), we used the approach of Meirmans (2019). For this, we calculated a test statistic *β*, which is the absolute value of the variable coefficient (“slope”) of an Analysis of Variance with either population (*β*_pop_) or ploidy level (*β*_ploidy_) as explanatory variable and the clustering results at *k* *=* 2 as response variable. This *β*-statistic is equivalent to calculating for every population/ploidy level the mean proportion of individuals assigned to the first cluster and then taking the absolute value of the difference between the two populations/ploidy levels. The values of the *β*-statistic can range from 0 to 1.

**Fig. 1:**
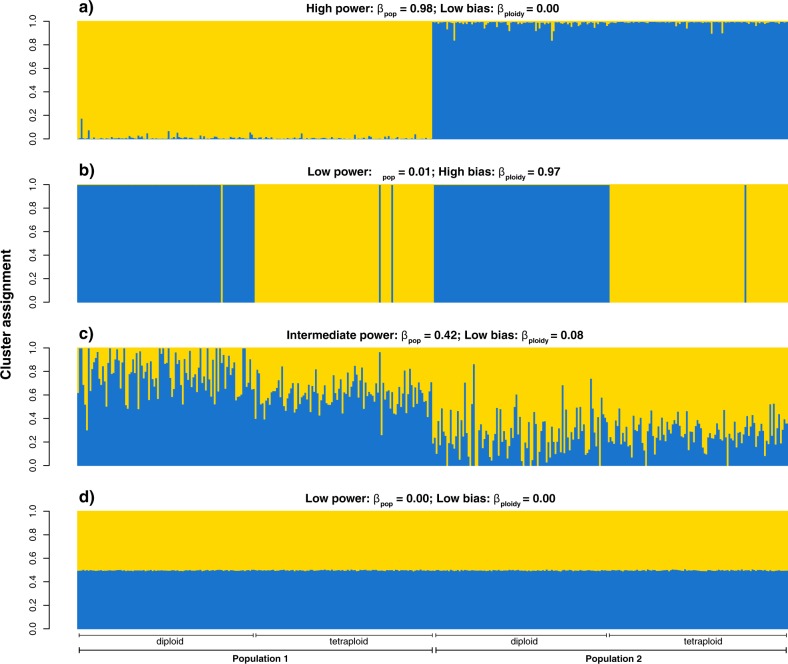
Example barplots showing different possible outcomes of the clustering analysis of mixed-ploidy data, with corresponding values for *β*_pop_ and *β*_ploidy_. **a** Structure plot showing result with correct clustering by population; co-dominant data with known dosage, *m* = 0.001. **b** *k*-means plot showing almost completely biased clustering by ploidy level; dominant data, *m* = 0.1. **c** Admixture plot showing intermediate power to cluster by population; co-dominant data with known dosage, *m* = 0.0065. **d** Structure plot showing absence of clustering; co-dominant data with known dosage, *m* = 0.1

If *β*_pop_ ≈ 1 and *β*_ploidy_ ≈ 0, this would indicate that the clustering analysis correctly identifies the two populations (Fig. 1a). If *β*_pop_ ≈ 0 and *β*_ploidy_ ≈ 1, this would indicate that the clustering analysis is biased, and identifies clusters by ploidy level, rather than by population (Fig. 1b). If both *β*_pop_ ≈ 0 and *β*_ploidy_ ≈ 0, no structure is detected, and corresponds neither to the populations nor to the ploidy levels (Fig. 1d). Of course, intermediate results are possible as well (Fig. 1c). However, it is logically impossible to simultaneously have both *β*_pop_ ≈ 1 and *β*_ploidy_ ≈ 1, because their sum cannot exceed 1.

## Results

### Varying strength of population differentiation

**Fig. 2:**
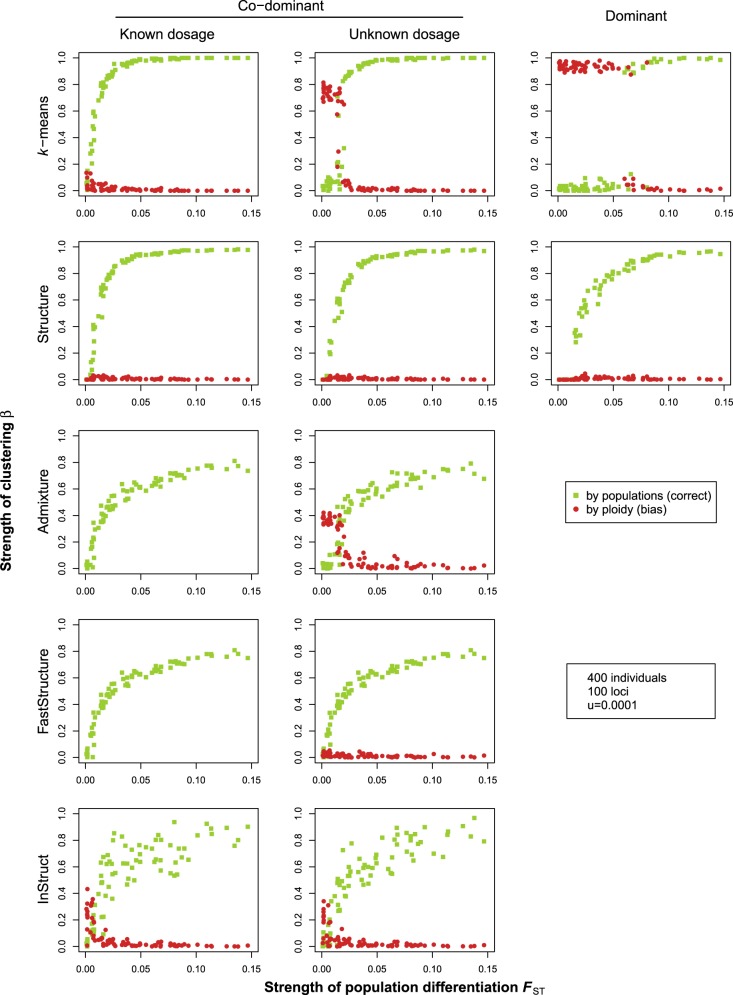
The degree of correct and biased clustering as a function of the simulated population differentiation (*F*_st_), for different clustering methods and three types of genetic data. The results are based on 10 replicate simulations for each of eight different migration rates, with 100 loci, a mutation rate of *u* = 0.0001, and 400 sampled individuals (100 diploids and 100 tetraploids from each of the two populations). Green dots show *β*_pop_, which indicates correct clustering by population; red dots show *β*_ploidy_, which indicates biased clustering by ploidy level. Note that for Admixture and FastStructure, bias cannot be calculated for co-dominant data with known dosage (hence no red dots shown)

For co-dominant markers with known dosage, we found hardly any spurious clustering by ploidy (i.e., no bias). The power to correctly detect the simulated structure differed between the different clustering methods. Structure and *k*-means were most powerful, with close to 100% of the runs correctly detecting the simulated structure for *F*_st_ > 0.05 (Fig. 2, first column). Only for weak simulated population differentiation (*F*_st_ < 0.05), there was a marked decrease in the power to correctly assign individuals to the populations (Fig. 2, first column). By contrast, Admixture and FastStructure only achieved up to 80% correct assignment to populations, even for the lowest simulated migration rates corresponding to *F*_st_ values up to 0.15. Finally, InStruct showed inconsistent results as indicated by the larger spread of the points compared to the other methods. This inconsistency derived from a lack of convergence among replicate runs of the algorithm. Furthermore, InStruct was the only method that showed a mild degree of spurious clustering by ploidy for co-dominant data, albeit only for lower *F*_st_ values.

For co-dominant markers with unknown dosage information, there was spurious clustering by ploidy for *k*-means, Admixture, and InStruct, mainly for cases where *F*_st_ < 0.025; Structure and FastStructure did not suffer from bias. Apart from this, the five methods differed in their power as before (Fig. 2, middle column).

For dominant markers, there was spurious clustering by ploidy for the *k*-means method for cases where *F*_st_ < 0.075. In other words, due to the strong bias at these levels of differentiation, there was virtually no power to correctly assign individuals to populations (Fig. 1, right column). Structure, the only other method that can deal with dominant data, did not show any spurious clustering, although its performance was somewhat reduced relative to that for co-dominant data.

With a lower mutation rate, there were no changes to the overall patterns, although the power to assign individuals correctly was somewhat reduced, partly due to the lower number of variable loci inherent to these simulations (Fig. S1). This effect was most pronounced for dominant data, where neither Structure nor *k*-means achieved 100% correct assignment even for the highest simulated *F*_st_ values. Perhaps counterintuitively, for methods that suffered from bias (clustering by ploidy) with our default parameter settings (for example *k-*means, Fig. 2), the reduced genetic variation associated with lower mutation rate also resulted in reduced bias (Fig. S1). This is because the bias has its origin in the unknown allele numbers in partial heterozygotes, which biases allele frequency estimates, and thus can lead to (incorrect) assignment by ploidy. With reduced genetic variation, there are simply less heterozygotes, and so there is less bias.

### Trading off the number of loci and individuals—weak population differentiation

**Fig. 3:**
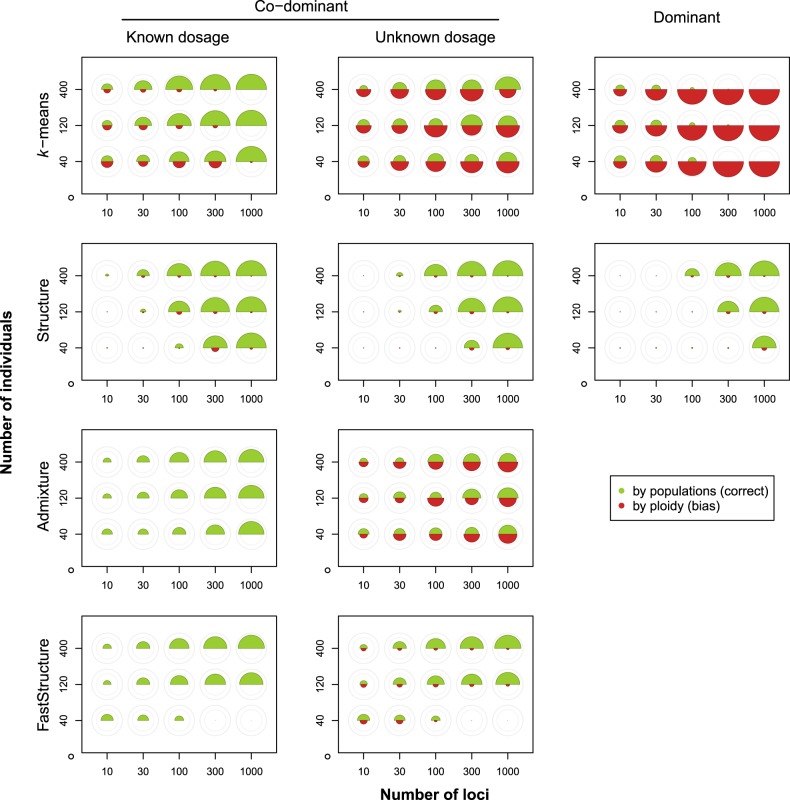
The degree of correct and biased clustering for different combinations of sample size and number of loci (averaged over ten replicate simulations per combination), under weak population differentiation (*m* = 0.01; *F*_st_ ≈ 0.016). The area of the upper (green) semicircles corresponds to the degree of correct clustering by population (*β*_pop_); the area of the lower (red) semicircles corresponds to the degree of biased clustering by ploidy level (*β*_ploidy_). The outer and inner light grey circles indicate *β*-values of 1.0 and 0.5, respectively. Note that for Admixture and FastStructure, bias cannot be calculated for co-dominant data with known dosage

For co-dominant markers with known dosage the power to correctly detect relatively weak simulated structure (*m* = 0.01; *F*_st_ ≈ 0.016) increased with the number of loci and individuals, for all methods (Fig. 3, first column). However, adding more loci was more effective to increase power than adding more individuals. In comparison to the other methods, Structure had lower power when the product of the number of individuals and loci was relatively low. The other three methods (*k*-means, Admixture, FastStructure) performed better in this range, but at the cost of increased bias; this was most pronounced for *k*-means clustering (Fig. 3, first column).

For co-dominant markers with unknown dosage, the power to correctly assign individuals under such weak differentiation was similar to that for data with known dosage for Structure and FastStructure (compare Fig. 3, left and middle column). However, *k*-means clustering and Admixture suffered from a marked increase in bias, and a reduction in power (Fig. 3, middle column). The extent of the bias increased with the number of loci, but not with the number of individuals sampled.

For dominant markers, Structure’s performance was similar as for the co-dominant markers, albeit with a somewhat reduced power (Fig. 3, right column). In contrast, *k*-means suffered strong bias; regardless of the number of loci, the bias was always stronger than the power to correctly assign individuals to the simulated populations. Adding more loci only made the situation worse, and adding more individuals did not matter much (Fig. 3, right column)

### Trading off the number of loci and individuals—strong population differentiation

**Fig. 4:**
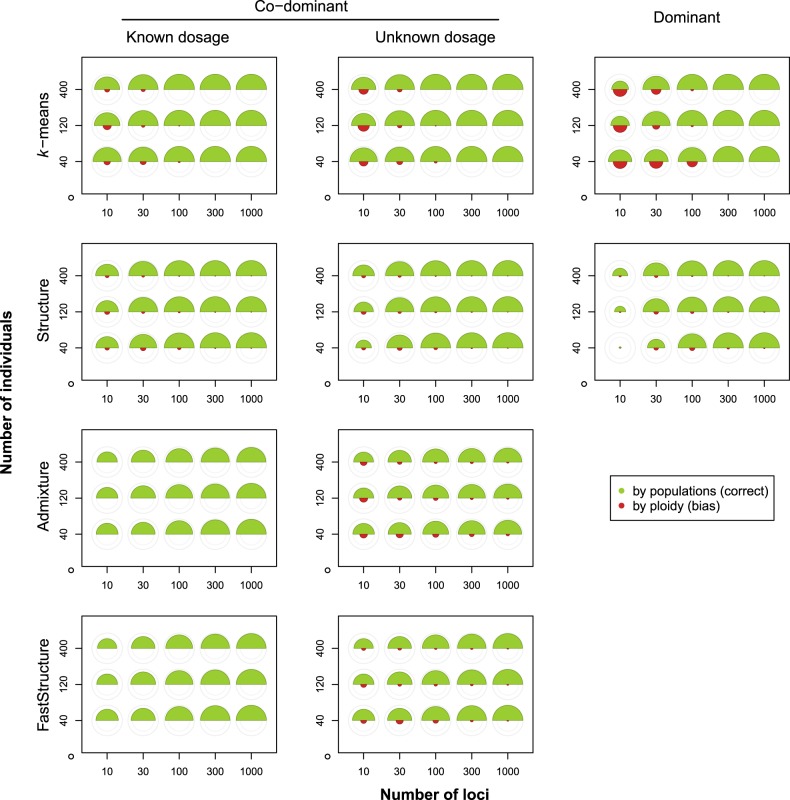
The degree of correct and biased clustering for different combinations of sample size and number of loci (averaged over ten replicate simulations per combination), under moderate population differentiation (*m* = 0.001; *F*_st_ ≈ 0.12). The area of the upper (green) semicircles corresponds to the degree of correct clustering by population (*β*_pop_); the area of the lower (red) semicircles corresponds to the degree of biased clustering by ploidy level (*β*_ploidy_). The outer and inner light grey circles indicate *β*-values of 1.0 and 0.5, respectively. Note that for Admixture and FastStructure, bias cannot be calculated for co-dominant data with known dosage

Generally, for all methods, there was reasonable power (Fig. 4) to correctly detect relatively strong simulated structure (i.e., moderate differentiation; *m* = 0.001; *F*_st_ ≈ 0.12). Most notably for *k*-means, some bias emerged for cases with low numbers of loci; this effect was most pronounced for the dominant markers. However, averaged over the ten replicate simulations, the bias never exceeded the power (Fig. 4).

### Simulated genotyping-by-sequencing data

**Fig. 5:**
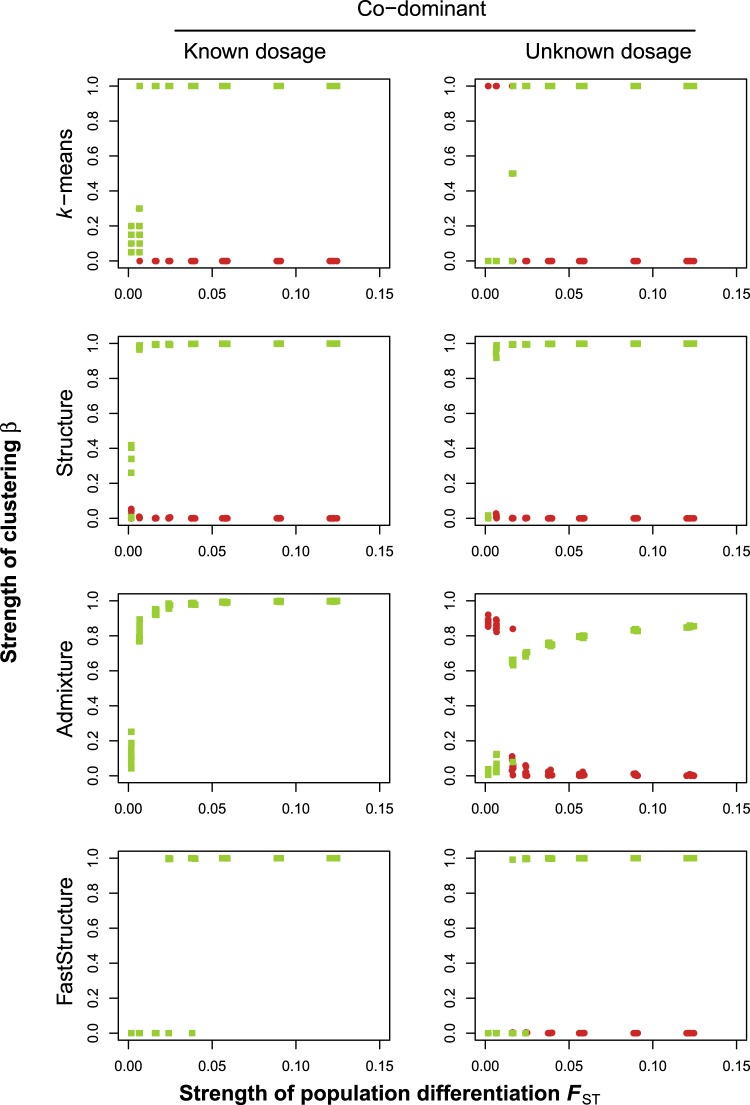
The degree of correct and biased clustering as a function of the population differentiation (*F*_st_), for simulated genotyping-by-sequencing data (10,000 loci, mutation rate of *u* = 0.0001, and 40 sampled individuals). Green dots show *β*_pop_, which indicates correct clustering by population; red dots show *β*_ploidy_, which indicates biased clustering by ploidy level. Note that for Admixture and FastStructure, bias cannot be calculated for co-dominant data with known dosage

For the simulated genotyping-by-sequencing data with 10,000 SNP loci and 40 sampled individuals, Admixture and FastStructure, the two methods that were specifically designed for such data, performed notably worse than the other two methods (Fig. 5). With known dosage, Structure and *k*-means always detected the correct population structure except when population differentiation was very low, whereas Admixture and FastStructure required slightly stronger differentiation. Interestingly, FastStructure sometimes failed to detect any structure even for an *F*_st_ value of about 0.04.

When allele dosage was unknown, *k*-means and Admixture again showed ploidy-related bias when population differentiation was low. In addition, Admixture also showed markedly lower performance in detecting the correct clustering by population.

## Discussion

We simulated genetic data for two mixed-ploidy populations with different degrees of differentiation to test the power of clustering methods to correctly infer the simulated population genetic structure and their risk of biased inference (spurious clustering by ploidy). The simulation model was purposely kept very simple to avoid processes (such as double reduction, a single origin of polyploidy, and pre- and postzygotic barriers) that would lead to allele frequency differences between the ploidy levels. Therefore, any differences between ploidy levels in the results of the clustering analyses must be due to bias. This allowed us to show that it is possible to infer the correct population structure without bias, even when marker information-content is reduced due to unknown dosage or dominant scoring. However, there were notable differences in power and risk of bias between marker types and between methods. These differences were especially obvious under scenarios of weak differentiation, where it is more difficult to detect the correct structure and there is thus more potential for biased inference. Below, we will discuss our findings for each of the methods (*k*-means clustering, Structure, Admixture, FastStructure, & InStruct). Based on this, we will conclude with the general recommendation to use the software Structure (Pritchard et al. 2000) for clustering analyses involving mixed-ploidy populations.

### *K*-means clustering

*K*-means is used either as a stand-alone analysis of population structure (Burnier et al. 2009; Kolar et al. 2012; Meirmans 2012; Trucchi et al. 2017) or within the framework of the DAPC method (Jombart et al. 2010), of which it is one of several steps. Even for large datasets, it takes only seconds to complete, and is free of assumptions regarding Hardy Weinberg and linkage disequilibrium (Jombart et al. 2010). One aspect in which *k*-means differs from the other methods is that it does not give estimates of the amount of admixture of individuals. Instead, it produces a “hard” clustering, where individuals are always assigned to a single population. This is much less computationally intensive than detecting admixture, and therefore *k*-means was much faster than Structure. However, this speed comes with a high risk of biased inference (spurious clustering by ploidy).

Biased inference with *k-*means clustering was especially likely for markers that have limited information, such as dominant markers and co-dominant markers with unknown dosage. Unfortunately, such markers are often chosen deliberately in systems with higher ploidy. For example, dominant markers have been advocated for polyploids for pragmatic reasons (e.g., Meudt and Clarke 2007), as many available population genetic tools did not work for polyploid co-dominant data, but did work for dominant data. This even led to the practice of reducing co-dominant data with unknown dosage into dominant data (Lepsi et al. 2009; Rodzen et al. 2004; Vallejo-Marin and Lye 2013), even though this involves an inherent loss of information due to the inability to detect heterozygosity (Dufresne et al. 2014). For analyses dealing with a single ploidy level, this approach may actually not result in a bias, as shown for parentage and sibship analysis (Wang and Scribner 2014). For analyses with mixed ploidy, on the other hand, biases are to be expected. For example, due to the inherent increased frequencies of band presence in higher ploidy levels, principal component analyses tend to cluster individuals of the same ploidy together (Meirmans et al. 2018).

Our findings illustrate that *k-*means clustering is indeed sensitive to the same issue, but it remains to be tested whether this affects conclusions from mixed-ploidy *k*-means analyses with dominantly scored data (e.g., Vallejo-Marin and Lye 2013). Moreover, we found that bias is not limited to dominant data, but –particularly given weak population differentiation– also appeared with co-dominant markers with unknown dosage (such as typical for microsatellites and low coverage sequencing approaches). Even for co-dominant data with known dosage (e.g., genotyping-by-sequencing methods with high coverage), bias arose with a low number of individuals or loci. For the inference of population structure of mixed-ploidy populations, *k*-means may therefore only be preferred over other methods for analysing data produced by genotyping-by-sequencing methods with sufficient coverage to allow obtaining full dosage information for many loci (1000 or more).

### Structure

Of all methods, Structure (Pritchard et al. 2000) provided the best power to correctly assign individuals to the simulated populations, with hardly any bias, even for dominant data or data with unknown dosage information. This is likely attributable to the fact that Structure, unlike any of the other methods, allows users to specify whether dosage information is missing or not. Only with rather weak population differentiation (*F*_st_ < 0.05) the power to detect structure decreased. This is in line with earlier simulation studies that have tested the power of Structure with diploid data (Evanno et al. 2005; Kalinowski 2011). However, specifically in comparison to *k*-means, Admixture and Fast-Structure, the required runtime of Structure is orders of magnitude longer (see methods for details). Furthermore, we noticed that it was not very clear from the Structure manual how the input-file should be formatted, especially for mixed-ploidy data (but see Meirmans et al. (2018) for a hopefully clearer description; also see the online supplements 3 and 4 for example datasets with haploid and correctly formatted input files, respectively). Correct formatting of the data is critical, especially for dominant markers: when we formatted these in a haploid presence/absence format, an often followed procedure (e.g., Durka et al. 2017), a very strong bias emerged (Fig. S2). Apparently, Structure is only able to correctly cluster dominant data for mixed-ploidy populations if the input file reflects the correct ploidy of each individual. Despite Structure’s sensitivity to input-file format and its relatively slow speed, other methods may only offer a viable alternative in some specific usage scenarios.

### Admixture & FastStructure

The development of Admixture (Alexander et al. 2009) and FastStructure (Raj et al. 2014) was driven by a need to efficiently analyse the increasingly large datasets generated with high-throughput sequencing methods. Both programs have in principle not been designed to deal with polyploid data. Thus, we could only use these programs for our purposes by subsampling to a diploid state (see methods for details), which meant that bias is by definition impossible for co-dominant data with known dosage (hence, bias is not plotted in the panels for Admixture & FastStructure in Figs. 2–5 and S1). For data with unknown dosage, bias is still possible due to missing information, and indeed appeared with weak structure. This was particularly pronounced for Admixture. Although there was less risk for bias with FastStructure than with Admixture, both methods had notably less power compared to Structure and *k*-means clustering. Both Admixture and FastStructure have been designed to deal with large SNP datasets, and indeed their performance much improves when more loci are added –though this could not resolve Admixture’s biased inference. However, even for the simulated genotyping-by-sequencing dataset with 10,000 SNPs, FastStructure was markedly inferior to Structure under weak population differentiation. Structure’s better power does come at a cost: running Structure on the same large datasets took several orders of magnitude longer than running Admixture or FastStructure; Structure took about half a day per replicate, whereas FastStructure took only a couple of seconds. Nevertheless, when the population divergence is expected to be low, Structure should be preferred over FastStructure, since the researcher’s patience is likely to be rewarded. Alternatively, for large datasets, FastStructure could be used for preliminary analyses with multiple *K*-values and replicates, followed by verification of a subset of the most promising partitions by Structure (e.g., Monnahan et al. 2019).

### InStruct

Of the five tested methods, InStruct (Gao et al. 2007) stood out both for being the only method that showed bias for co-dominant data and for providing the most inconsistent clustering results. There generally was a lack of convergence across independent replicates with different seeds for the random number generator. This suggests that a large number of replicates –many more than the ten replicates that we used– should be run to make sure that the overall best result will be obtained. Unfortunately, running InStruct was very slow, taking more than 10 times as long for a single replicate as Structure. Because of its poor performance and its long runtime, we only ran InStruct for our standard parameter set of 100 individuals and 100 loci (Fig. 2), and not for the expanded parameter sets (other figures). Despite these drawbacks, there is one argument in favor of InStruct. Unlike any of the other methods, InStruct can explicitly deal with inbreeding, which may be useful for polyploids as these can show a higher rate of self-fertilisation than related diploids (Barringer 2007). However, although we did not test this option in InStruct, it seems unlikely that the program’s performance will improve given the more complex task of simultaneously detecting inbreeding and population structure.

## Conclusions and recommendations

Correct inference of population structure with mixed-ploidy populations critically depends on the choice of method, the degree of population differentiation and to some extent on the type of marker. Using simulated data, we compared the power and potential bias of *k-*means clustering and the model-based approaches implemented in Structure, Admixture, FastStructure and InStruct. Our results showed that all methods performed reasonably well under scenarios of strong population differentiation. However, as the degree of differentiation is usually not known *a priori*, it is safer to use methods that also perform well when population differentiation is weak. In this light, Structure clearly outperformed the other methods, with its derivative FastStructure as the closest contender. The latter may provide a reasonable alternative to Structure in cases where computation times become prohibitive due to data volume and population differentiation is not expected to be weak. Although *k-*means clustering should generally be avoided for markers with incomplete genotype information, it is more powerful than FastStructure for large datasets (1000 or more loci) with known dosage. Future studies should test whether the biases we identified become more pronounced with unequal sample sizes (Puechmaille 2016). Given our findings, it would also be useful to re-analyse published mixed-ploidy datasets and verify whether findings may have been affected by any of the potential biases we identified.

### Data archiving

The code used for simulations and our analyses are available from the Dryad Digital Repository: 10.5061/dryad.6g635f6.

## Supplementary information

Fig S1

Fig S2

Supplement 3

Supplement 4

Data Set 1
