# Supplementary material for: Structure is more robust than other clustering methods in simulated mixed-ploidy populations
Source: Heredity (Edinb). 2019 Jul 8;123(4):429–41. doi: 10.1038/s41437-019-0247-6 (PMC6781132; doi:10.1038/s41437-019-0247-6)
Supplement: Supplementary file 1 — Fig S1 [file 41437_2019_247_MOESM1_ESM.pdf]

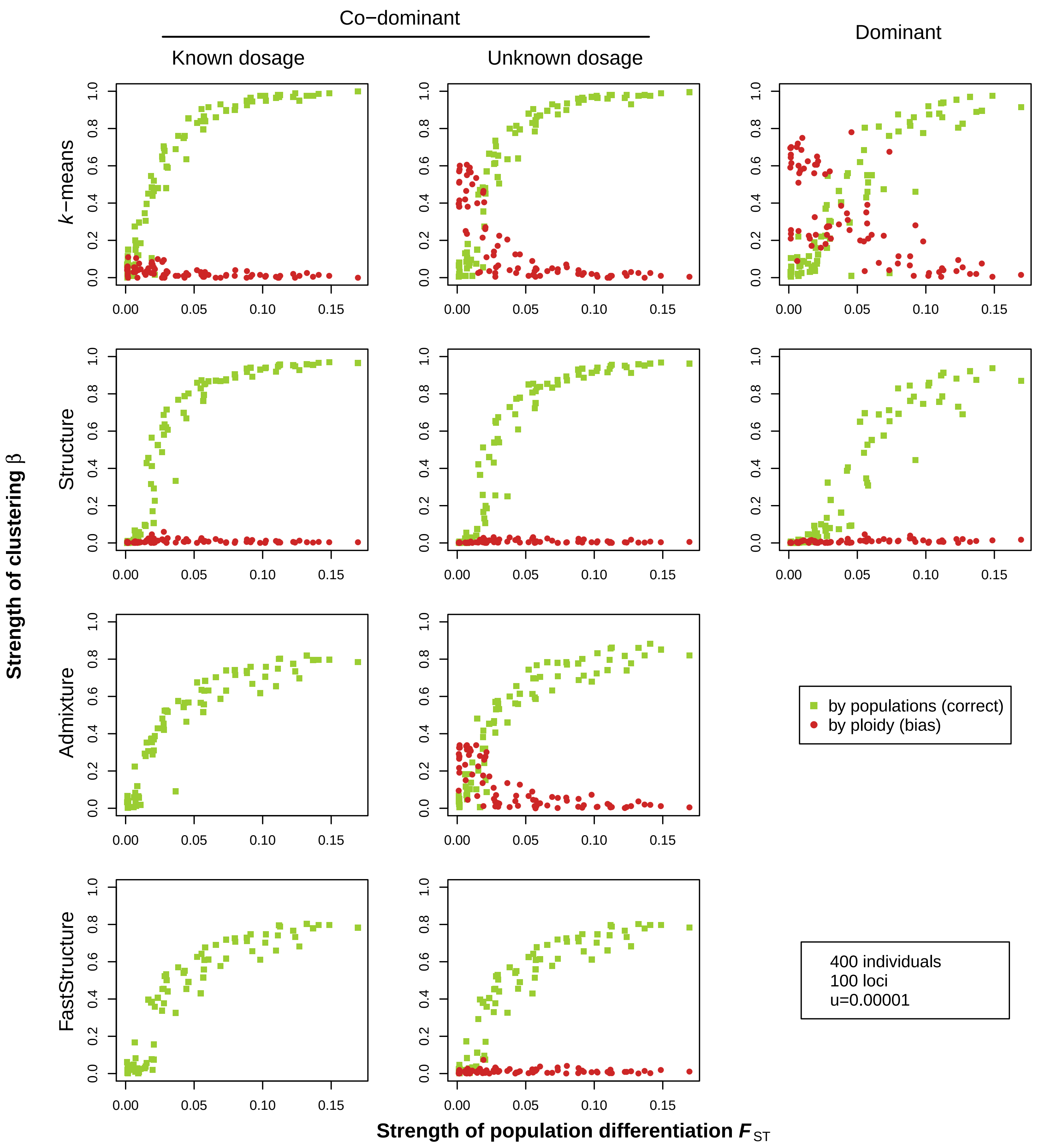

Co−dominant
Known dosage
Unknown dosage
Dominant
k−means
Structure
Admixture
FastStructure
Strength of clustering β
Strength of population differentiation F_{ST}
0.0
0.2
0.4
0.6
0.8
1.0
0.00
0.05
0.10
0.15
by populations (correct)
by ploidy (bias)
400 individuals
100 loci
u=0.00001
