# Supplementary figures and images for: Structure is more robust than other clustering methods in simulated mixed-ploidy populations
Source: Heredity (Edinb). 2019 Jul 8;123(4):429–41. doi: 10.1038/s41437-019-0247-6 (PMC6781132; doi:10.1038/s41437-019-0247-6)

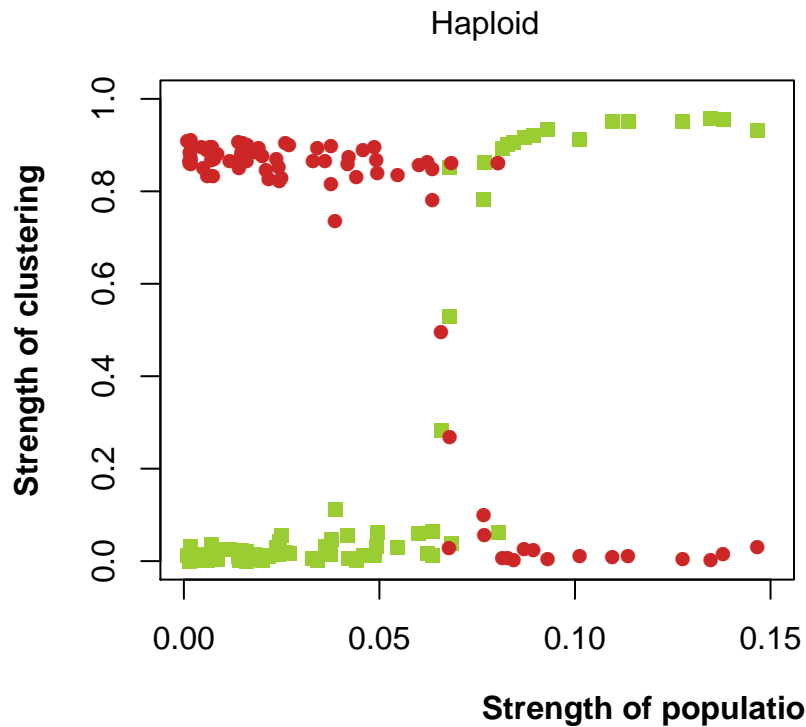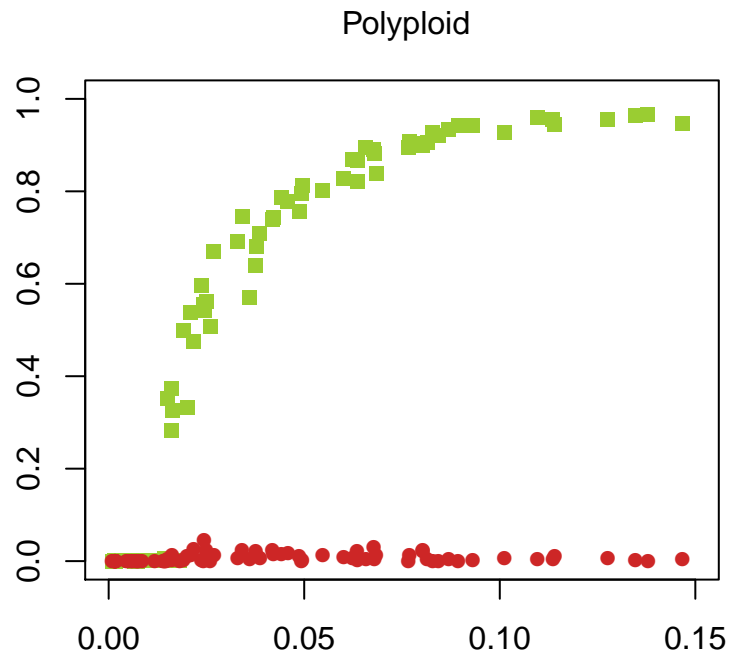

Supplement: Supplementary file 2 — Fig S2 [file 41437_2019_247_MOESM2_ESM.pdf]
